# Impact of academic majors on entrepreneurial intentions of Vietnamese students: An extension of the theory of planned behavior

**DOI:** 10.1016/j.heliyon.2021.e06381

**Published:** 2021-03-08

**Authors:** Trung Kien Dao, Anh Tuan Bui, Thi Thu Trang Doan, Ngoc Tien Dao, Hieu Hoc Le, Thi Thu Ha Le

**Affiliations:** a Faculty of Economics and Business, Phenikaa University, Yen Nghia, Ha Dong, Ha Noi, Viet Nam; b Foreign Trade University, 91 Chua Lang, Lang Thuong, Dong Da, Ha Noi, Viet Nam; c School of Economics and Management, Hanoi University of Science and Technology, 01 Dai Co Viet, Bach Khoa, Hai Ba Trung, Ha Noi, Viet Nam

**Keywords:** Academic majors, Business students, Engineering students, Entrepreneurial intentions, Theory of planned behavior

## Abstract

This article investigates the effect of academic majors on entrepreneurial intentions of engineering and business students. The research model was established based on the extension of the theory of planned behavior (TPB) through combining the TPB model, perceived risks, academic majors and personalities of students. A sample of 1844 students from the four largest universities in engineering and business in Vietnam were surveyed. The main findings indicated that (i) the relationship in the TPB model was accepted except the effect of subjective norms on entrepreneurial intentions; (ii) perceived risks have negative impacts on perceived behavioral control; (iii) male engineering students have a higher entrepreneurial intentions than female students, but this result was not found in business students; (iv) engineering students have a higher entrepreneurial intentions than business students; (vi) there are no differences between the entrepreneurial intention of students coming from rural and urban areas. The study also contributes to some policy discussion to extend the current debate about the role of academic majors that students take in university in the entrepreneurial process as well as the importance of entrepreneurial students to society.

## 1 Introduction

Entrepreneurship promotion has been increasingly discussed by Vietnamese policy-makers in recent years. Entrepreneurial activity has been seen as an engine for long–term national economic development (Romer, 1994). Entrepreneurship motivation to create a dynamic enterprise community helps to shape the new growth engine for the economy, which is now a priority of the Vietnamese government. In Vietnam's economy, small and medium-sized firms are estimated to have created approximately 50% GDP and nearly 90% new jobs annually over the last several decades (VCCI, 2018). With the slogan "Tectonic government", the Vietnamese government has issued several policies aiming to reinforce entrepreneurial activities such as the national program "Supporting Innovative Startup Ecosystem until 2025″ and the “Start-up nation” goal (Decision 844/QĐ-TTg of 2016). As university students are expected at the core of the new entrepreneurial generation, the Ministry of Education and Training has launched a project named “Supporting student start-ups until 2025”, according to the Program 217/KH-BGĐT (The Ministry of Education and Training, 2019). The project's objective is to promote and facilitate entrepreneurship and business incubation activities of students nationwide.

Entrepreneurship is a process of creativity and innovation where there is a potential to add value to products, create job opportunities, raise productivity, revitalize and diversify markets, improve social welfare, and further, economic development (Guerrero et al., 2008; Urbano et al., 2017; Esfandiar et al., 2019). Entrepreneurship of an individual is considered as changing the mindset and self-empowering to promote economic development by employment creation and global economic integration (Hisrich and Peters, 2012). Entrepreneurship often leads to innovation and development (Drucker, 1999). Entrepreneurship is of great importance to developing countries as they promote economic growth and innovative capacity in many industries (Ribeiro – Soriano, 2017). Job creation, economic development, and poverty reduction are usually the main benefits in entrepreneurship (Willis, 2011; Ribeiro – Soriano, 2017). Thus, to assure a continuous source of new entrepreneurs to the economy, scholars and policymakers should be aware of the entrepreneurial intentions of potential entrepreneurs as well as factors that encourage their entrepreneurship.

To encourage the entrepreneurship spirit, it is necessary to understand human decision making (Autio et al., 2001). Therefore, many studies have been conducted with a focus on the concept of entrepreneurial intentions. Entrepreneurial intentions are the first step in the process of business formation and are often intentional (Engle et al., 2010) because entrepreneurship is a predictable activity (Krueger and Carsrud, 1993). Accordingly, entrepreneurial intentions are used to predict future entrepreneurial behaviors (Krueger et al., 2000a, Krueger et al., 2000b; Linan and Chen, 2009). Several scholars emphasize that the stronger the entrepreneurial intentions are, the higher the chances new business activities are formed (Botsaris and Vamvaka, 2016; Kautonen et al., 2015).

Numerous studies on entrepreneurship focused on the concept of entrepreneurial intentions with the assumption that, firstly, entrepreneurial intention is an essential step in the foundation of an organization; secondly, entrepreneurship is mostly intentional (Engle et al., 2010) because business is what people plan to do (Krueger and Carsrud, 1993). So, the entrepreneurial intentions have been proved to be the main factor that foresees future entrepreneurial behaviors (Krueger et al., 2000a, Krueger et al., 2000b; Botasris and Vamvaka, 2016). Entrepreneurial intentions play a central role in the entrepreneurship process because it is the starting stage and the incentive of entrepreneurship that encourages individuals to start their new businesses (Krueger and Carsrud, 1993). Recent research has recognized the importance of the models based on entrepreneurial intentions because they include theoretical perspectives as well as directions to explain the determinants of entrepreneurial intentions (Autio et al., 2001; Krueger et al., 2000a, Krueger et al., 2000b; Linan and Chen, 2009; Maresch et al., 2016; Guerrero et al., 2008; Esfandiar et al., 2019; Nguyen et al., 2019).

The important role of entrepreneurship intention is confirmed in words of Krueger et al., 2000a, Krueger et al., 2000b that entrepreneurial intentions signal how intensely one is prepared and how much effort one is planning to commit to carrying out entrepreneurial behavior. Due to the importance of this topic, research on entrepreneurial intentions is conducted quite frequently (Esfandiar et al., 2019; Fayolle and Linan, 2014). However, no specific study reconciles alternative model into a single one. Some authors indicated the compatibility of intention-based models (Boyd and Vozikis, 1994; Krueger et al., 2000a, Krueger et al., 2000b; Fayolle and Linan 2014). The theory of planned behavior (Ajzen, 1991) is seen as the most common approach, which is most relevant and provides predominant specification (Fayolle and Linan 2014). The theory of planned behavior isconsidered as a key theoretical ground of entrepreneurial intentions study; however, researches carried out in different contexts can be put together to create a better development model based on TPB in the system of theory about entrepreneurial intentions (Fayolle and Linan 2014). For example, the research of Forlani and Mullins (2000) found that perceived risk affected the attitude toward entrepreneurship. Findings of some research have also confirmed the similar result showing attitude isaffected by perceived risk (Hmieleski and Corbett, 2006; Van Gelderen et al., 2008; Zhang et al., 2015). According to Fayolle and Linan (2014), there are five ways to expand TPB model including (i) methodological and theoretical issues; (ii) influence of personal-level variables on entrepreneurial intentions; (iii) entrepreneurship education and intention; (iv) the role of context and institution; and (v) the entrepreneurial process and the intention-behavior link.

Currently, there have been quite few studies on entrepreneurial intentions of science and engineering students (Phan et al., 2009; Yanez et al., 2010; Maresch et al., 2016). Meanwhile, the role of entrepreneurship in developing technology is becoming more important, because their entrepreneurial activities create new, high-quality firms (Åstebro et al., 2012) and drive up economic growth (Guerrero et al., 2008; Ribeiro – Soriano, 2017; Esfandiar et al., 2019). Promoting technology-based entrepreneurship may be vital, especially for areas affected by an economic crisis (Heitor et al., 2014; Maresch et al., 2016). Therefore, several scholars call for more studies among different disciplines, especially in science and technology (Rauch and Hulsink, 2015). In this research, we focus on investigating engineering students because they are the most likely to start-up technology-oriented ventures (Maresch et al., 2016), compared with business students who are provided with more business and start-up knowledge from the universities.

Although studies on entrepreneurial intentions are widely implemented, scholars are still debating about new and better research models to explain entrepreneurial intentions in the new context (Fayolle and Linan 2014). It is because entrepreneurship could have different meanings among different research contexts such as its historical, institutional, spatial and social circumstances (Welter, 2011; Fayolle and Linan 2014). Therefore, this research focuses on developing of a new model, which is the combination of TPB model and perceived risk and student characteristics (gender, family job and place of residence) to explain the constructs impacting on entrepreneurial intentions as well as to make a comparison between the model for engineering students and that for business students. The paper begins with the literature review on entrepreneurial intentions, perceived risks, the elements of the TPB model and control variables (gender, family job, academic majors and place of residence). In the next part, the article presents a research methodology and research results. Finally, the article discusses the findings and suggests several recommendations to promote the entrepreneurial intentions of engineering and business students in Vietnam.

## 2 Theoretical framework and hypotheses

### 2.1 Entrepreneurial intentions

The entrepreneurial intentions have become a great matter of concern in the field of entrepreneurship study (Fayolle and Linan 2014). Longitudinal research by Kautonen et al. (2015) confirms that entrepreneurial intentions may foresee entrepreneurial behaviors of individuals. Thus, the question of what influences entrepreneurial intentions involves policymakers, scholars, and practitioners. Entrepreneurial intentions are defined as the self-belief of individuals who intend to start a new business and plan to do it in the future (Thompson, 2009). Entrepreneurial intentions are either a plan or a desire to create new companies or business activities (Krueger et al., 2000a, Krueger et al., 2000b) or start an organization (Popescu et al., 2016). Entrepreneurial intentions and behaviors are closely related (Kautonen et al., 2015; Esfandiar et al., 2019; Neneh, 2019). To be more specific, entrepreneurial behavior is the process of searching, evaluation, and exploitation of business opportunities to make new business activities (Shane and Venkataraman, 2000) with entrepreneurial intentions being the very first and very important step of the whole process.

**Figure 1:**
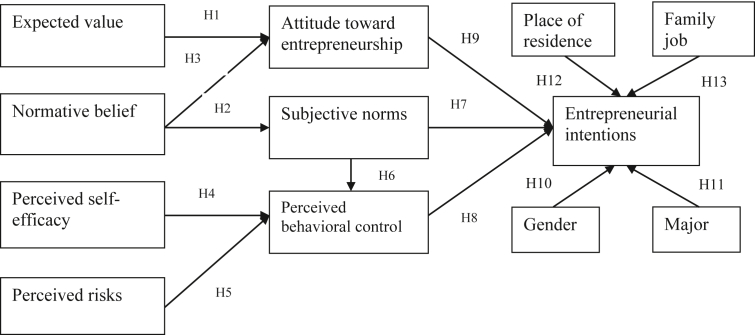
Proposed model.

The Theory of Planned Behaviors (Ajzen, 1991) is one of the most well-known theories about entrepreneurial intentions (Fayolle and Linan 2014; Maresh et al., 2016). Entrepreneurial intentions are a planned activity; "planned" consists of different levels rather than a simple yes or no (Thompson, 2009). Strong intention encourages behaviors, especially planned behaviors (Ajzen, 1991), so it is considered as a determinant of behaviors (Fayolle and Lassas-Clerc, 2006). The TPB model, which has become the fundamental theory of many studies on entrepreneurial intentions, is widely adopted by scholars worldwide with different little modifications (Krueger et al., 2000a, Krueger et al., 2000b; Linan and Chen, 2009; Urbano et al., 2017; Maresch et al., 2016; Esfadinar et al., 2019; Nguyen et al., 2019). The combination of entrepreneurial intentions research with students' majors has been examined in a few previous studies (Autio et al., 2001; Linan, 2004; Luthje and Franke, 2003). In this research, we established the research model combining the TPB model with perceived risk as well as students' contextual conditions (gender, academic majors, place of residence and family job) like the control variables. The proposed model is described in Figure 1.

### 2.2 Hypotheses

#### 2.2.1 Expected value

The expected value of individuals is the desire and feeling of individuals about their competence or ability to execute an activity or task (Ajzen and Madden, 1986; Krueger et al., 2000a, Krueger et al., 2000b). The expectation is a psychological variable displaying feelings about competence and desire to perform important tasks (Krueger et al., 2000a, Krueger et al., 2000b). For entrepreneurial activities, the expectation of individuals about their entrepreneurial competence affects their attitude towards entrepreneurial activities. Individuals with high expectations often have a positive attitude towards jobs, plans or schedules to do important things (Kautonen et al., 2015). In other words, higher individual expectation leads to a more positive attitude towards work which is specific entrepreneurship in the case of entrepreneurial activities. Therefore, the hypothesis is presented below.

H1 Expected value has a positive impact on attitude toward entrepreneurship of students.

#### 2.2.2 Normative beliefs

Normative beliefs are defined as self-beliefs affected by members of society (Ajzen, 1987; Ajzen and Fishbein, 1980; Krueger et al., 2000a, Krueger et al., 2000b). Normative belief is a cognitive variable displaying the ability of social groups to affect individuals' decision making. Normative beliefs may influence individuals' decision making because individuals often refer to the opinions of surrounding people including family, friends, colleagues, and intimate people. The Theory of Planned Behavior explains that normative beliefs affect and form the subjective norms of individuals, and the ideas may affect the completion of an activity or duty (Ajzen 1987, 1991; Krueger and Kickul, 2006; Kautonen et al., 2010). Apart from subjective norms, normative beliefs also influence the attitude towards activities (Olds et al., 2005; Reynolds, 1997). The extent to which individuals are affected by others influences their attitude. Therefore, normative beliefs develop perceived subjective norms and attitudes toward tasks that are related to personal bias. They may either encourage or hinder individuals from executing an activity. Therefore, this research proposes a hypothesis:

H2 Normative beliefs have a positive impact on the subjective norms of students.

H3 Normative beliefs have a positive impact on the attitude towards entrepreneurship of students.

#### 2.2.3 Perceived self-efficacy

Perceived self-efficacy is the awareness of the competence to perform business activities, the strength of the belief in becoming a business owner (Boyd and Vozikis, 1994; Prabhu et al., 2012; Krueger et al., 2000a, Krueger et al., 2000b). Perceived self-efficacy displays individuals' awareness of their ability and competence to do something and promotes an optimistic self-view in the pursuit of goals (Barbosa et al., 2007). Perceived self-efficacy is measured by the ability to establish, maintain, control, and realize opportunities (Linan and Chen, 2009) or the ability to solve problems and develop business ideas (Autio et al., 2001; Kickul and Gundry, 2002). Perceived self-efficacy indicates how confident individuals are in finishing a task successfully. The perception of individuals is also related to collective activities, which means the feelings about the competence of themselves, of their group/team whose members may perform equivalent tasks (Esfadinar et al., 2019). The sense of self-competence is closely related to entrepreneurial intentions. Experimental researches also confirm that high perceived self-efficacy has a positive impact on perceived behavior control (Krueger et al., 2000a, Krueger et al., 2000b; Autio et al., 2001; Linan and Chen, 2009; Shepherd and Krueger, 2002; Esfadinar et al., 2019). Therefore, a hypothesis is proposed as below:

H4 Perceived self-efficacy has a positive impact on perceived behavior control of students.

#### 2.2.4 Perceived risks

The perceived risk factor is considered in our proposed intention construct because entrepreneurial activities are risky endeavors by nature, and examining risk is a central part of entrepreneurial intentions (Zhang et al., 2015). As business involves many risks, the personal trait that sets out a clear difference between an entrepreneur and an employee is the ability to take risks (Drucker, 1999). Risk describes a preference for uncertainty with a distribution of possibilities over certainty. In the definition of Liñán and Chen (2009), perceived risks are a personal viewpoint on unstable incidents. Hmieleski and Corbett (2006) concluded that start-up decisions were influenced by two elements and one of them is one's thinking about risks (perceived risks) in new venture creation. Research has shown that a positive attitude toward risk or a willingness to bear uncertain results is associated with entrepreneurial intentions. In contrast, individuals with a high perceived risk had weaker levels of entrepreneurial intentions. Van Gelderen et al. (2008) concluded that a higher perceived market risk implies a higher chance of failure of nascent activities. Autio et al. (2001) underscored the impact of students' perceptions of risks on entrepreneurship as negatively influencing student intention towards envisaging an entrepreneurial career. This aspect is, however, not included in the original theory of planned behaviors. By adding these important factors in our suggested model, we intended to verify if young people's attitude towards risk has a negative role in influencing their entrepreneurial intentions. So, the hypothesis is developed as follows:

H5 Perceived risks have a negative impact on the perceived behavior control of students.

#### 2.2.5 Subjective norms

Subjective norms may be understood as the effect of perceived social pressure on the performance of one action (Ajzen 1991). Subjective norms refer to the opinion or belief of one's close people such as family members, close friends, or of those who may influence the decision to or not to carry out one action. The influence exercised by these people may have an impact on the intention to become an entrepreneur or to perform entrepreneurial activities that can be beneficial (Armitage and Conner, 2001). Subjective norm is a cognitive variable that refers to the perception of the initiative, the recognition of opportunities, or the acknowledgment of actions and the means to perform actions (Autio et al., 2001). Subjective norms may play a part in changing one's perception as to whether one should perform or not perform one action. In other words, the subjective norm affects one's behavior control. This relationship has been upheld in some recent studies which show the direct positive impact of subjective norms on perceived behavior control (Hsu, 2012; Usman and Yenita, 2019). Some studies examined the effects of subjective norms on the attitudes of individuals (Ajzen, 1991). However, studies in the entrepreneuship sector often did not consider this relationship as a causal relationship (Krueger et al., 2000a, Krueger et al., 2000b; Zhang et al., 2015; Maresch et al., 2016). Meanwhile, many studies have shown that subjective norms have a direct influence on the intention to act (Krueger et al., 2000a, Krueger et al., 2000b; Autio et al., 2001; Zhang et al., 2015; Usman and Yenita, 2019; Maresch et al., 2016). Therefore, this study put forward the following hypotheses:

H6 Subjective norms have a positive impact on the perceived behavior control of students.

H7 Subjective norms have a positive impact on the entrepreneurial intention of students.

#### 2.2.6 Perceived behavioral control

Perceived behavioral control is defined as the individual's perception of the ease or difficulty of acting (Ajzen, 2002). In terms of entrepreneurship, PBC is considered as the perception of the success of becoming an entrepreneur. It is, therefore, a concept quite similar to self-efficacy and perceived feasibility (Shapero and Sokol, 1982). All three concepts refer to the sense of capacity regarding the fulfillment of firm-creation behaviors. PBC would include not only the feeling of being able but also the perception about the control ability of the behavior. High PBC will have an impact on the attitude towards start-up (Devonish et al., 2010), motivation or intention of entrepreneurship so that it pushes the desire and determination of an individual to start their business (Krueger et al., 2000a, Krueger et al., 2000b). Therefore, this study proposes the following hypothesis:

H8 Perceived behavior control has a positive impact on the entrepreneurial intentions of students.

#### 2.2.7 Attitude towards entrepreneurship

In the TPB model, attitude and behavior reflect the feelings of individuals about the excitement, plan, or willingness to take part in and have positive opinions about the activity (Ajzen, 1987). For students' entrepreneurial activities, the attitude towards entrepreneurship indicates the positiveness or readiness to take part in entrepreneurial activities when opportunities arise (Krueger et al., 2000a, Krueger et al., 2000b). A positive attitude towards entrepreneurship of individuals may also be recognized by the preference and desire to own a business rather than to be hired (Tella and Issa, 2013). Attitude towards entrepreneurship is also associated with the assessment of advantages and disadvantages (Maresch et al., 2016). Individuals who are more positive about the results of entrepreneurship have higher positiveness about entrepreneurial activities; hence, stronger entrepreneurial intentions (Krueger et al., 2000a, Krueger et al., 2000b; Autio et al., 2001; Segal et al., 2005; Van Gelderen and Jansen, 2006; Franke and Luthje, 2004; Linan and Chen, 2009; Maes et al., 2014). Thus, the research proposes the following hypothesis:

H9 The attitude towards entrepreneurship has a positive impact on the entrepreneurial intentions of students.

#### 2.2.8 Control variables

Based on several previous studies (Maes et al., 2014; Maresch et al., 2016; Neneh, 2019; Nguyen et al., 2019) we contend that some characteristics of students may influence their entrepreneurial intentions.

*Gender*

Traditionally, business was dominated by men (Ahl, 2006). Therefore, a smaller number of female entrepreneurs leads to fewer available female role models to impact the entrepreneurial intentions of women (Maes et al., 2014). The Global Entrepreneuship Monitor (GEM) survey indicated that the percentage of start-up entrepreneurs who are male is often higher than female (Monitor, 2018, 2019). This perception is even further consolidated in Confucian countries such as Vietnam, where the role of men in the professional world is more respected than that of women. Traditions and prejudices have long ascribed family roles rather than professional roles to women, which leads to the fact that our society sees fewer women in the business world than men. Consequently, society does not have many strong female entrepreneurs in business compared to men. This is likely to lead to less female motivation to become entrepreneurs than men (Barnir et al., 2011; Shinnar et al., 2012). This is also verified in some previous studies that showed that men have higher entrepreneurial intentions than women (Caro - Gonzales et al., 2017; Maresch et al., 2016; Neneh, 2019; Nguyen et al., 2019). Therefore, we speculate that female students have lower entrepreneurial intentions than male and therefore hypothesize:

H10 Male students have a higher entrepreneurial intention than female students.

*Academic majors*

A major can also influence a student's entrepreneurial intentions because the knowledge provided can affect perceptions of business opportunities or confidence in managing a business. The argument that entrepreneurship is easily stimulated, nurtured, and thrived by education has been supported both within and outside the academic world (Marques et al., 2012). Traditionally, business major students are more systematically equipped with management, business and entrepreneurial knowledge than those in other disciplines. This can lead to the perceptions of opportunities, and attitudes toward entrepreneurship being different between business students and students of other disciplines. However, studies on the relationship between entrepreneurship education and initial intentions do not have unanimous conclusions about this relationship. Some studies have shown the effect of entrepreneurship and professional education on entrepreneurial intentions (Fayolle and Lassas-Clerc, 2006; Marques et al., 2012; Maresch et al., 2016), some other studies did not confirm this relationship (Rodrigues et al., 2012). In this study we predict that business students have higher entrepreneurial intentions than engineering students. Therefore, the next proposed hypothesis is:

H11 Students in business majors have a higher entrepreneurial intention than engineer students

*Place*

The place of residence can also influence a student's intention to start a business due to factors of socio-economic circumstances. Nisthantha (2009) emphasized that the difference in living area is an important factor affecting the entrepreneurship of young people due to differences in socio-economic or political conditions. Students may have a better sense of socioeconomic and political conditions that will likely support their entrepreneurial intentions, and vice versa (Ozaralli and Rivenburgh, 2016; Kolosta et al., 2018). In Vietnam, nearly 70% of the population live in rural areas and more than 50% of students come from rural areas (General Statistics Office of Viet Nam - GSO, 2019). Rural areas with lower socioeconomic conditions tend to have lower levels of business dynamism than densely populated urban areas. Such living conditions can affect their perception of business opportunities. Therefore, we assume that students from rural backgrounds have lower levels of dynamism due to a less dynamic economic life. Therefore, students from rural areas have lower spirits toward entrepreneurship than students from urban areas. As a result, we propose the following research hypothesis:

H12 Students coming from urban areas have a higher entrepreneurial intention than students from rural areas.

*Family job*

Family background is also considered as one of the factors influencing students' intention to start a business. Research by Crant (1996) shows that growing up in business families has a significant impact on individuals to start a business of their own. Children of entrepreneurs can learn the factors involved in running a business and consider starting a new business as a natural choice (Cooper et al., 1994). Family background plays an important role in the entrepreneurial development of members (Alsos et al., 2011), parents can be role models for children's decisions (Mueller, 2006). Research by Chaudhary (2017) confirms that the background of a self-employed family has a positive effect on an individual's business intentions. These arguments suggested that students from business families may have more exposure to business activities and start a business. Over time, family conditions affect the personality and perception of the members with start-up activities. Therefore, we speculate that students from business families have higher entrepreneurial intentions, and the suggested hypothesis is:

H13 Students having a business family have a higher entrepreneurial intention than students with a family of other occupations.

## 3 Methodology

### 3.1 Development of the survey instrument

We used a survey method to collect the data and test the proposed hypotheses through a structured questionnaire. The items in each construct were developed based on literature review and adapted from previous papers about behavioral intentions and entrepreneurial intentions. In which, expected value construct was measured by five items adapted from Krueger et al., 2000a, Krueger et al., 2000b. Attitude towards entrepreneurship construct was evaluated by five items based upon from Krueger et al., 2000a, Krueger et al., 2000b, Linan and Chen (2009). Subjective norms construct was measured by four items adapted from Krueger et al., 2000a, Krueger et al., 2000b, Autio et al. (2001) and Kolvereid (1996). Normative beliefs construct was measured by four items referenced form Autio et al. (2001), Krueger et al., 2000a, Krueger et al., 2000b and Linan and Chen (2009). Perceived self-efficacy was evaluated by seven items adapted from Autio et al. (2001), Krueger et al., 2000a, Krueger et al., 2000b and Linan and Chen (2009). Perceived behavioral control construct was measured by six items that are developed form Krueger et al., 2000a, Krueger et al., 2000b and Linan and Chen (2009). Perceived risks construct was measured by four items adapted from Hisrich and Peters (2012), and Linan and Chen (2009). Entrepreneurial intentions construct was measured by five items based upon from Krueger et al., 2000a, Krueger et al., 2000b and Linan and Chen (2009).

Survey data was collected in 2017–2018 from students who are studying engineering and business in Vietnam. The items were translated from English into Vietnamese and using the back translation method to ensure the reliability and concordance of the translation process. The questionnaire was adjusted through discussing with ten experts in entrepreneurship in some universities (Hanoi University of Science and Technology, Foreign Trade University and National Economics University) and pilot test with 50 engineering students and 54 business students. The items of each construct in the research model were described in Table 5. All items were measured by a five-point Likert scale, anchored by 1: strongly disagree and 5: strongly agree.

### 3.2 Sample and data collection

The population was identified as students who were studying in engineering and business in Vietnam. There are two samples conducted to collect: (1) preliminary sample and official sample. In the preliminary sample, we used a convenient survey to collect data at Hanoi University of Science and Technology. In order to ensure privacy rules and research ethics, we kept the respondents anonymous on the questionnaire. As a result, we distributed 150 questionnaires and gained 138 valid questionnaires for the preliminary evaluation of the scale. In the official sample, we used a stratified sampling combined cluster sampling method. The four largest engineering and business universities were chosen to distribute survey in both the North (Hanoi) and the South (Ho Chi Minh city) including Hanoi University of Science and Technology, Ho Chi Minh City University of Technology, National Economics University and Foreign Trade University (both Hanoi and Ho Chi Minh City campus). The sub-samples were taken according to the average number of students enrolled in the period 2015–2017 at each university.

Official data were collected with the support of lecturers at the universities chosen. The questionnaires were sent to lecturers who agreed to support our survey according to the allocation norms for each campus. As a result, with 2,500 distributed we gained 1,844 valid questionnaires from two groups of engineering students and business students, the response rate reached 74%. The characteristics of students were described in Table 1.

**Table 1:** Characteristics of survey students.

Categorical		Frequency (%)
Gender	Male	987 (53.5%)
	Female	857 (46.5%)
Year	Freshman	119 (6.5%)
	Sophomore	253 (13.7%)
	Junior	459 (24.9%)
	Senior	584 (31.7%)
	Five-year student	429 (23.3%)
Major	Business	855 (46.4%)
	Engineering	989 (53.6%)
Place of residence	Urban	825 (44.7%)
	Rural	1019 (55.3%)
Family job	Agriculture	659 (35.7%)
	Office	476 (25.8%)
	Business	533 (28.9%)
	Other	176 (9.5%)

### 3.3 Common method and non-response bias

Since we used only one survey method to collect the data, common method bias can influence the validity of research conclusions (Podsakoff et al., 2003) or the true relationship between constructs produces parameter bias estimation (Malhotra et al., 2007). Thus, we used some of the ways to reduce the effect of common method bias. According to the suggestion of Podsakoff et al. (2003), first, we used an anonymous method with respondents to collect data, the items were carefully designed to avoid ambiguous items. The items were also reflected positively or negatively to control for acquiescence and dis-acquiescence biases (Podsakoff et al., 2012). After the collection data process, we use Harman's test to evaluate common method bias in data collected. The result of the test indicated that when fixed to a unique factor of all items, the total variance explained was smaller than 50% (30.127%). This proves that common method bias did not affect the results of the study.

Non-response is a problem survey research and affects the research results. In this study, to examine non-response bias, we use the t-test to compare early respondents and late respondents divided at a ratio of 70:30 (Armstrong and Overton, 1977) on categorical variables (academic majors, place; gender). The finding found no difference between the two groups (p-value > 0.05). These test results suggested that response bias was not a concern in our research.

### 3.4 Data analysis method

We used multivariate data analysis method to analyze the data collected. Confirmatory factor analysis was used to evaluate the reliability and validity of each construct in the model. The criteria to consider the model achieve overall fit with actual data when CFI, GFI, TLI, and IFI all are greater than 0.9, and RMSEA is less than 0.08 (Hair et al., 2010; Kline, 2011; Hooper et al., 2008). The factor loadings of items within each construct are greater than 0.5, showing that the constructs in the model achieve convergent validity. The constructs achieve reliability when composite reliability (CR) and Cronbach's Alpha are greater than 0.6, average variance extracted (AVE) is greater than 50% (Hair et al., 2010; Nunnally and Burstein, 1994). To test discriminant validity between constructs in the model, we used criteria comparing the square root value of AVE and correlation coefficients in the model (Hair et al., 2010) or using a 95% confidence interval of correlation coefficients (Anderson and Gerbing, 1988). If the square root of AVE values of each construct is greater than the correlation of constructs, or the 95% confidence interval of the correlation coefficient does not contain one value indicating that the constructs reach discriminant validity. We used structural equation modeling to test hypotheses with criteria statistically significant at a level of 5%. The negative suppression phenomenon is also considered in this study if there is a difference in the sign of the beta coefficients and the sign of the correlation between constructs in the model (Maassen and Bakker, 2001).

## 4 Findings

### 4.1 Preliminary evaluation of the scale

To evaluate the preliminary reliability of each construct, we used Cronbach Alpha's coefficient and exploratory factor analysis (EFA) with preliminary data (n = 138). The result showed all research constructs meet the requirement of internal consistency and were unidimensional scale. All Cronbach's Alpha coefficients were greater than 0.6 and the corrected – item-total correlation of items in each construct was greater than 0.3. EFA analysis result indicated that all KMO coefficients were greater than 0.5, p-value (Bartlett's test) < 0.05 and total variance explained were greater than 50% and factor loadings of items in each construct were greater than 0.5 (Table 2).

**Table 2:** The Preliminary reliability of each construct (n = 138).

Constructs	Cronbach's Alpha	Corrected-item total correlation minimum	KMO	p-value	Factor loading minimum	TVE (%)
EXP	0.782	0.394	0.744	0.000	0.567	55.255
ATT	0.843	0.375	0.806	0.000	0.512	62.615
BEL	0.764	0.369	0.753	0.000	0.569	60.606
SUB	0.747	0.446	0.608	0.000	0.404	61.129
SEF	0.754	0.369	0.814	0.000	0.695	53.552
PBC	0.790	0.447	0.744	0.000	0.633	54.087
RIS	0.702	0.415	0.644	0.000	0.629	54.062
INT	0.879	0.638	0.866	0.000	0.762	67.423

*Notes:* EXP is expected value, ATT is the attitude towards entrepreneurship, BEL is normative beliefs, SUB is subjective norms, SEF is perceived self-efficacy, PBC is perceived behavioral control, RIS is perceived risks and INT is entrepreneurial intentions.

### 4.2 Official measurement scale test

#### 4.2.1 Reliability and validity

We used confirmatory factor analysis (CFA) to test the property of our measures with the saturated model (final model). The results analysis showed that the model achieved overall fits with the actual data: CFI = 0.950; GFI = 0.951; TLI = 0.938; IFI = 0.950, all were larger than 0.9 and RMSEA = 0.046 was less than 0.08. All the constructs had factor loadings higher than the benchmark level of 0.05, which indicated that the constructs achieved convergent validity. The Cronbach's alpha and composite reliability coefficients of all constructs exceed the 0.7 benchmarks, and all AVEs were larger than 0.5 (Table 3). These tests showed that our measures for constructs have achieved internal consistency and reliability.

**Table 3:** Reliability, convergent validity, and model fit index.

Constructs	N of items	Cronbach's Alpha	Range of loadings (CFA)	Composite Reliability	Average variance extracted
EXP	5	0.735	0.699–0.851	0.753	0.606
ATT	5	0.793	0.710–0.775	0.783	0.547
BEL	4	0.755	0.684–0.783	0.771	0.530
SUB	5	0.741	0.704–0.847	0.753	0.607
SEF	7	0.793	0.664–0.783	0.779	0.541
PBC	6	0.792	0.670–0.780	0.772	0.531
RIS	4	0.766	0.616–0.806	0.765	0.524
INT	5	0.870	0.738–0.806	0.873	0.580

Model fit index: CFI = 0.950; GFI = 0.951; TLI = 0.938; IFI = 0.950, RMSEA = 0.046.

#### 4.2.2 Discriminant validity

The analysis result indicated that most constructs have the square root of AVEs greater than the correlation coefficients between the construct and any other construct except the pair ATT – INT (0.803 and 0.740). However, using the bootstrap method to estimate the 95% confidence interval of the correlation coefficients showed that the confidence interval of the largest correlation was the pair ATT – INT did not contain 1 value (0.778 ÷0.831). On the other hand, all constructs used in the model achieved discriminant validity (Table 4).

**Table 4:** Discriminant validity of constructs in the mode.

Constructs	EXP	ATT	BEL	SUB	SEF	PBC	RIS	INT
EXP	**0.779**							
ATT	0.371	**0.740**						
BEL	0.215	0.515	**0.728**					
SUB	0.029	0.196	0.395	**0.779**				
SEF	0.473	0.078	0.148	-0.065	**0.736**			
PBC	0.432	0.544	0.453	0.215	0.468	**0.729**		
RIS	0.186	-0.024	0.052	-0.053	0.506	0.149	**0.724**	
INT	0.472	0.803	0.396	0.129	0.291	0.608	0.103	**0.762**

*Notes:* Diagonal elements (bold) are the square roots of AVEs.

#### 4.2.3 The difference of degree operationalization in each construct among student groups

The descriptive statistics and t-test analysis result suggest that there are slight differences between engineering students and business students in terms of entrepreneurial intentions and constructs which influence entrepreneurial intentions in the proposed model. The findings indicated that items in constructs such as EXP, ATT, BEL and INT, engineering students have a higher degree. In contrast, the items in constructs such as SUB, SEF, PBC and RIS, business students have a higher degree (Table 5).

**Table 5:** Operationalization.

Items	Mean(SD)			Diff
	Full sample (n = 1844)	Engineering Students (n = 989)	Business Students (n = 855)	
*Expected values*				

I know how to develop an entrepreneurship project	2.323(0.974)	2.372(0.985)	2.267(0.958)	-0.105∗
I have prepared to set up an enterprise	2.111(1.050)	2.161(1.073)	2.054(1.021)	-0.107∗
If I try to set up an enterprise, it will be successful	3.169(1.024)	3.264(1.031)	3.058(1.005)	-0.205∗∗
I think I know how to identify opportunities	3.294(0.898)	3.410(0.890)	3.161(0.890)	-0.248∗∗
I can solve arising problems	3.382(0.835)	3.442(0837)	3.312(0.828)	-0.130∗
*Attitude towards entrepreneurship*				
I am interested in becoming an entrepreneur	3.457(1.135)	3.595(1.138)	3.297(1.111)	-0.297∗∗
If I had an opportunity and necessary resources, I would start-up	3.816(1.071)	4.023(1.035)	3.577(1.062)	-0.447∗∗
I want to become an entrepreneur if I have to choose my career	3.728(1.457)	3.771(1.107)	3.678(1.778)	-0.093
Becoming an entrepreneur would bring me great satisfaction	3.670(1.048)	3.714(1.046)	3.620(1.050)	-0.094
Becoming an entrepreneur would bring more advantages than disadvantages	3.460(1.031)	3.442(1.067)	3.481(0.988)	0.039
*Normative beliefs*				
My friend would support my start-up idea	3.490(0.937)	3.485(0.922)	3.495(0.956)	0.009
Family members would support my entrepreneurship idea	3.494(0.996)	3.515(0.988)	3.470(1.004)	-0.044
My classmates would support my entrepreneurship idea	3.339(0.918)	3.440(0.895)	3.222(0.931)	-0.218∗∗
People surrounding me thinks that it is admirable to become an entrepreneur	3.433(0.962)	3.424(0.993)	3.443(0.925)	0.020
*Subjective norms*				
At myuniversity people are encouraged to actively pursue their ideas	3.711(1.045)	3.565(1.088)	3.880(0.967)	0.314∗∗
At university, I have the opportunity to meet many people who have good ideas to start up	3.607(1.026)	3.420(1.061)	3.825(0.938)	0.405∗∗
I think that entrepreneurship is trainable	3.645(0.954)	3.659(0.975)	3.628(0.928)	-0.031
I know many students at myuniversity who have run a start-up successfully	3.433(1.045)	3.244(1.062)	3.651(0.981)	0.408∗∗
At my university, there are many support activities for student to create an innovative start-up business	2.898(1.022)	2.581(0.964)	3.264(0.963)	0.683∗∗
*Perceived self-efficacy*				
Starting a business would be easy for me	2.012(0.994)	1.866(0.914)	2.182(1.054)	0.317∗∗
Maintaining the value of a business is not too difficult	2.153(0.999)	2.068(0.945)	2.251(1.051)	0.184∗∗
I can control a start-up	2.786(1.046)	2.798(0.931)	2.773(1.165)	-0.025
Start-up would bring me more opportunities to develop	3.413(0.976)	3.494(1.007)	3.318(0.931)	-0.176∗∗
I know the necessary aspects to begin a business	2.614(0.970)	2.570(0.959)	2.665(0.980)	0.095∗
Only unexpected events would hinder me from starting up	2.510(1.009)	2.467(1.013)	2.560(1.003)	0.093∗
Developing a business idea would be easy for me	2.309(1.017)	2.279(1.015)	2.343(1.019)	0.064
*Perceived behavioral control*				
If I start-up, my enterprise would sustain and develop	3.115(0.874)	3.191(0.862)	3.027(0.879)	-0.164∗∗
I think my start-up would be highly successful	3.045(0.884)	3.080(0.887)	3.005(0.879)	-0.075
I think I have sufficient traits to become an entrepreneur	3.059(0.985)	3.087(1.016)	3.026(0.947)	-0.061
Knowledge and experiences motivate me to become an entrepreneur	2.914(1.039)	2.807(1.072)	3.037(0.985)	0.231∗∗
I have a network of relationship that supports me when I start-up	2.738(1.048)	2.661(1.040)	2.827(1.049)	0.166∗∗
I can easily access supporting information for entrepreneurship	2.832(1.017)	2.772(1.031)	2.901(0.998)	0.128∗∗
*Perceived risks*				
I think that success in life is not based on my ability	2.415(1.176)	2.298(1.208)	2.550(1.124)	0.251∗∗
I think my life is most predetermined by powerful people	2.334(1.112)	2.203(1.089)	2.484(1.119)	0.281∗∗
I think that my success is mainly due to luck	2.401(1.038)	2.273(0.988)	2.549(1.074)	0.276∗∗
I think success in entrepreneurship is mainly due to luck	2.415(1.027)	2.293(1.026)	2.556(1.011)	0.262∗∗
*Entrepreneurial intentions*				
I am ready to do everything to become an entrepreneur	3.050(1.043)	3.101(1.045)	2.991(1.039)	-0.110∗
My objective is to become an entrepreneur	3.183(1.096)	3.187(1.127)	3.178(1.061)	-0.009
I will try my best to start and manage my company	3.547(1.075)	3.651(1.098)	3.427(1.035)	-0.224∗∗
I will surely start my own business shortly (ie: right after graduating)	2.908(1.076)	2.903(1.059)	2.913(1.097)	0.011
I have a big will about my start-up	3.174(1.121)	3.291(1.112)	3.037(1.117)	-0.254∗∗

*Notes:* ∗p < 0.05; ∗∗p < 0.01.

#### 4.2.4 Structural model and hypotheses test

We conducted six structural equation modeling analyses to test hypotheses in the model (two models for the full sample, two models for engineering students sample and two models for business students sample). The analysis result presented that the model received an acceptable fit to the actual data: CFI, GFI, TLI, and IFI all were larger than 0.9 and RMSEA was smaller than 0.08. In the first model (model a) we found that SUB had the negative direct effect on INT (β < 0, p-value < 0.01), while the correlation of SUB and INT is positive (r > 0, p-value < 0.01). This could be caused by the negative suppression phenomenon between the constructs in the model (Maassen and Bakker, 2001). In addition, SUB has positive correlation with PBC (r > 0, p-value < 0.01). Therefore, we may conclude that the SUB has an indirect effect on INT via PBC. In the second model (model b), after eliminating the relationship which did not have statistically significant (p-value > 0.05) and direct relationship between SUB and INT, the analysis result indicated that the model achieved the overall fit to the actual data: CFI, GFI, TLI, and IFI all were larger than 0.9 and RMSEA was smaller than 0.08, and the model fit indexes were improved (Table 6). The main findings from data analysis were summarized below:

**Table 6:** Result of SEM analysis (standardized coefficient).

Relationships	Full sample (n = 1844)		Engineering students (n = 989)		Business students (n = 855)	
	Model 1a	Model 1b	Model 2a	Model 2b	Model 3a	Model 3b
(H2) BEL→SUB	0.420∗∗ (12.935)	0.411∗∗(12.551)	0.363∗∗(7.745)	0.389∗∗(7.536)	0.570∗∗(12.554)	0.552(12.128)
(H1) EXP→ATT	0.307 ∗∗(10.837)	0.313∗∗(11.020)	0.336∗∗(8.156)	0.476∗∗(8.201)	0.265∗∗(7.054)	0.278(7.320)
(H4) SEF→PBC	0.843∗∗(15.730)	0.854∗∗(15.762)	0.715∗∗(12.163)	0.788∗∗(12.161)	0.824∗∗(9.324)	0.861(9.346)
(H5) RIS→PBC	-0.354∗∗(-8.075)	-0.360∗∗(-8.145)	-0.311∗∗(-6.634)	-0.328∗∗(-6.653)	0.171∗(-2.328)	-0.195(-2.542)
(H6) SUB→PBC	0.266∗∗(8.625)	0.240∗∗(8.001)	0.247∗∗(5.911)	0.239∗∗(5.780)	0.394∗∗(8.547)	0.338(7.667)
(H3) BEL→ATT	0.439∗∗(14.908)	0.428∗∗(14.629)	0.292∗∗(7.392)	0.367∗∗(5.256)	0.623∗∗(13.786)	0.604(13.541)
(H9) ATT→INT	0.682∗∗(24.502)	0.666∗∗(24.359)	0.720∗∗(19.382)	0.669∗∗(19.465)	0.656∗∗(15.034)	0.585(14.533)
(H8) PBC→INT	0.392∗∗(14.434)	0.358∗∗(14.359)	0.340∗∗(10.134)	0.340∗∗(10.098)	0.461∗∗(10.067)	0.426(10.477)
(H10) Gender→INT	0.046∗(2.347)	0.052∗(2.647)	0.055∗(2.376)	0.118∗(2.558)	0.026(0.960)	-
(H11) Major→INT	-0.075∗∗(-3.810)	-0.049∗(-2.507)	-	-	-	-
(H7) SUB→INT	-0.107∗∗(-4.590)	-	-0.068∗(-2.216)	-	-0.178∗∗(-4.480)	-
(H12) Place→INT	-0.017(0.968)	-	-0.006(-0.279)	-	-0.018(-0.697)	-
(H13) Family job→INT	-0.029(-1.704)	-	-0.051∗(-2.219)	-0.114∗(-2.293)	-0.012(-0.485)	-
Model fit index	CFI = 0.903; GFI = 0.919; TLI = 0.887; IFI = 0.904; RMSEA = 0.055	CFI = 0.922; GFI = 0.932; TLI = 0.908; IFI = 0.923; RMSEA = 0.053	CFI = 0.920; TLI = 0.906; IFI = 0.921; GFI = 0.926; RMSEA = 0.052	CFI = 0.928; TLI = 0.916; IFI = 0.928; GFI = 0.931; RMSEA = 0.050	CFI = 0.924; TLI = 0.910; IFI = 0.925; GFI = 0.921; RMSEA = 0.051	CFI = 0.924; TLI = 0.911; IFI = 0.924; GFI = 0.918; RMSEA = 0.056

*Notes:* ∗p < 0.05; ∗∗p < 0.01.

The values in parentheses are critical ratios: (Family job: 1 = business, 0 = others; Place: 1 = Rural, 0 = Urban; Gender: 1 = Male, 0 = Female; Major: 1 = Business, 0 = Engineering).

The hypotheses that were accepted in the models (a full sample, a sample of engineering students and a sample of business students) include the effect of BEL on SUB, EXP, and BEL on ATT, SEF, SUB and RIS on PBC; ATT and PBC on INT. However, we did not find the direct impact of SUB on entrepreneurial intentions. In other words, while hypotheses H1, H2, H3, H4, H5, H6, H8, and H9 were accepted, H7 was rejected.

There were differences of degree of entrepreneurial intentions between female students and male students in engineering students, not business students. Specifically, male engineering students have a higher entrepreneurial intention than female students, while this result was not found in business students. On the other hand, part of the hypothesis H10 was accepted.

The findings indicated that there were differcences in the level of entrepreneurial intentions between engineering students and business students. In fact, the result showed that engineering students have a higher entrepreneurial intentions than business students (β = -0.075 < 0, p-value < 0.05), which was against our initial expectations. Thus, hypothesis H11 was rejected.

Residential areas students, before they enter university, did not have an impact on their entrepreneurial intentions in the three models (a full sample, a sample of engineering students, and a sample of business students). It is to say that hypothesis H12 was rejected.

The family's professional background has a weak impact on their entrepreneurial intentions. Specifically, the impact of their parents' careers is found only in the engineering students group, not in the business student. In contrast to our expectations, students who have a family background related to business and commerce have lower entrepreneurial intentions than those in other groups. In other words, the result verified a part of hypothesis H13.

**Figure 2:**
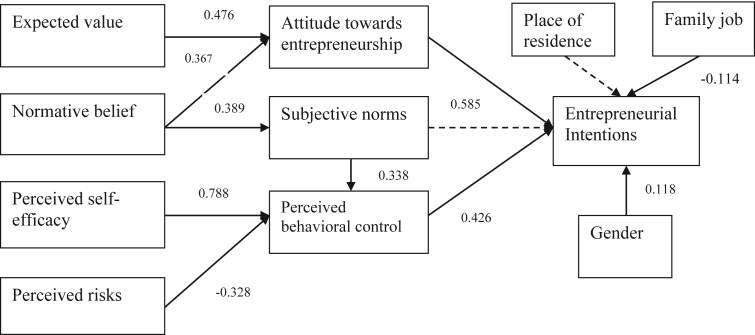
The relationship between the constructs in the model of engineering stutdents sample. *Notes*: Dotted lines are not statistically significant.

**Figure 3:**
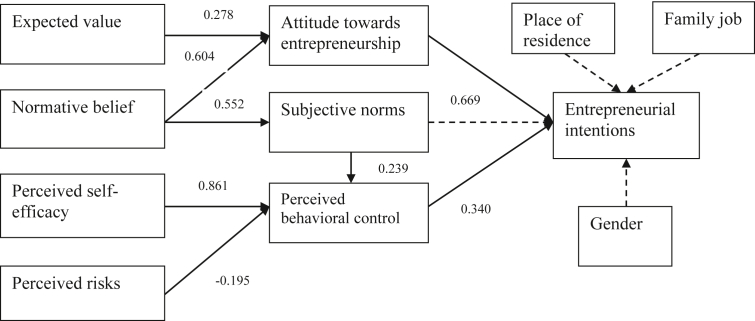
The relationship between the constructs in the model of business students sample. *Notes*: Dotted lines are not statistically significant.

The result also indicated that the impact of constructs in the TPB model and perceived risks on entrepreneurial intentions are different between engineering students and business students (see Figure 2 and Figure 3).

The INT is affected not only directly by PBC and ATT but also indirectly by EXP, SUB, BEL, SEF, and RIS. Thus, to assess the effect of each factor on INT we used the direct, indirect and total effect coefficients in the path analysis with the bootstrapping method (n = 1000). The estimation result was shown in Table 7, in which the highest effect comes from ATT (0.666), followed by PBC (0.358), BEL (0.321), SEF (0.305), EXP (0.208), RIS (0.129).

**Table 7:** The effect of the constructs on INT in the model.

Dependent variable	Effect	Major	Gender	RIS	SEF	BEL	EXP	SUB	PBC	ATT
SUB	Direct	0.000	0.000	0.000	0.000	0.411	0.000	0.000	0.000	0.000
	Indirect	0.000	0.000	0.000	0.000	0.000	0.000	0.000	0.000	0.000
	Total	0.000	0.000	0.000	0.000	0.411	0.000	0.000	0.000	0.000
PBC	Direct	0.000	0.000	-0.360	0.854	0.000	0.000	0.240	0.000	0.000
	Indirect	0.000	0.000	0.000	0.000	0.099	0.000	0.000	0.000	0.000
	Total	0.000	0.000	-0.360	0.854	0.099	0.000	0.240	0.000	0.000
ATT	Direct	0.000	0.000	0.000	0.000	0.428	0.313	0.000	0.000	0.000
	Indirect	0.000	0.000	0.000	0.000	0.000	0.000	0.000	0.000	0.000
	Total	0.000	0.000	0.000	0.000	0.428	0.313	0.000	0.000	0.000
INT	Direct	-0.049	0.052	0.000	0.000	0.000	0.000	0.000	0.358	0.666
	Indirect	0.000	0.000	-0.129	0.305	0.321	0.208	0.086	0.000	0.000
	Total	-0.049	0.052	-0.129	0.305	0.321	0.208	0.086	0.358	0.666

## 5 Discussion and implications

### 5.1 Discussion

We conducted this study intending to evaluate the impact of socio-psychological constructs on entrepreneurial intentions of engineering and business students. We developed a framework that is expanded theory of planned behavior (TPB) combined with the perceived risk construct and contextual variables as well as personal characteristics. The findings showed that there are differences in entrepreneurial intentions between engineering students and business students as well as between male students and their female counterparts.

Conventionally, business students are perceived to have a higher entrepreneurial intention than engineering students (Kautonen et al., 2015; Maresch et al., 2016). However, our data showed that this assumption is not true for Vietnamese students and may not be true for students in other emerging countries. Our result of nearly 2000 students showed that, in contrast, engineering students have a higher entrepreneurial intention than business students. This finding reaffirms the need to consider contextual and personal characteristics of the student in studies of entrepreneurial intentions (Kautonen et al., 2015; Maresch et al., 2016; Fayolle and Linan, 2014).

Differences in our findings should be interpreted in the study context. The fact that the entrepreneurial intentions of engineering students is higher than those of business students may derive from the proliferation of success stories of science and technology start-ups in media recently. Vietnamese successful start-ups in Vietnam such as MoMo (fin-tech), Luxstay (short-term office rental connection), Haravan (online business solutions) or Wefit (connecting customers with gym centers) are related to science and technology. Through media outlets, examples of successful entrepreneurship in science and technology may enhance young people's confidence in opening new businesses, business students are better informed of business systems, start-ups, project management or identification of business opportunities. It is this knowledge that may make business students more cautious with entrepreneurship opportunities, which explains why their intention to start a business is lower.

An interesting result in our study is that engineering students give a higher score in the aspects related to self-awareness such as expected values, attitude towards entrepreneurship, normative beliefs, perceived risks and entrepreneurial intentions than business students. Meanwhile, the aspects related to risk, external influences such as subjective norms, perceived self-efficacy and perceived behavioral control business students get higher scores. This showed that engineering students tend to be more confident in their capability, while business students are more cautious about external environmental aspects. The differences in curriculum courses between the two groups may be a reason for this phenomenon. In Vietnam, engineering programs often focus on specialized knowledge but not entrepreneurship-related subjects. The lack of business knowledge may lead to their overconfidence state, which may explain why engineering students have higher entrepreneurial intentions. Our interview with two start-up training experts from the FTU Innovation and Incubation Space of Foreign Trade University (FISS) and the Entrepreneurship and Innovation Center of Hanoi University of Science and Technology (BKUP) further strengthened our hypothesis that the better understanding of business knowledge may reduce optimism about opening new business of students. Experts noted that the readiness for opening new business of students who take part in start-up training courses often decreases after they complete the course.

*“…Normally, students are very optimistic about the idea of starting a new business and their intention to entrepreneurship before our entrepreneurial training courses. However, at the end of the course, they are usually more careful and take a closer look at all aspects of the start-up environment. When they know more about entrepreneurship, they become more cautious about the risk related to entrepreneurship actions” (the expert from FISS).*

*“…Engineering students in our training courses are often very confident in their product ideas before the course. But often they become timider when they know more about the entrepreneurial ecosystem and the tasks they need to do to start a new business successfully” (the expert from BKUP).*

We call the phenomenon (reduced confidence when understanding business knowledge much) is "the effect of reducing confidence by awareness". These findings are similar to the Dunning – Kruger effect in psychology (Kruger and Dunning, 1999; Aqueveque, 2018; Gibbs et al., 2017). It means that individuals who do not understand much about a problem may become overconfident beyond their comprehension.

We found a significant effect of the gender on entrepreneurial intentions, in which male students have higher entrepreneurial intentions than female ones in engineering students group. This reinforces our initial assumption that in Vietnam, under the influence of Confucianism which emphasizes the social role of men than that of women, consequently has a lack of female role models in business. Besides, the prejudice that emphasizes the family's role over the social role of women may impact the perception that a successful woman is a dedicated housewife rather than a powerful female entrepreneur. Several previous studies have pointed out that although the role of women is increasingly enhanced in society, the business field is still dominated by men (Ahl, 2006). These reasons may dampen women's motivation to become entrepreneurs (Barnir et al., 2011; Shinnar et al., 2012). The traditional perception in Confucian society about the family role of women and the scarcity of powerful female entrepreneurs are two main reasons explaining why female students have lower entrepreneurial intentions.

We found no difference in entrepreneurial intentions by place of residence before attending college and by the occupation of the family. There seems to be no difference in entrepreneurial intentions between students from rural and urban areas although there is quite big a difference in business activities between the two regions. This may be explained by the fact that business activities do not have much impact on students before they go to college. The university entrance exam in Vietnam is a very tough one and students who want to be admitted to prestigious universities must spend a lot of time on exam preparation. Consequently, they tend to be less interested in other social activities like business activities. In contrast to our expectations and quite surprisingly, students from a business-oriented family show no higher entrepreneurial intentions than others. This may stem from the historical traces of Vietnamese economic institutions. Before 1986, Vietnam was a country with a centrally-planned economy based on the Soviet model. Private economic activities were illegal and would receive severe punishment (Le, 2018). This resulted in the fact that businessman has only reappeared in the last 30 years. This may be a reason that students from different family backgrounds have no different degrees of entrepreneurial intentions.

As expected, we found that perceived risk has a quite strong negative impact on students' entrepreneurial intentions. This finding is also supported by many previous studies showing that individuals who perceive a higher level of risk tend to have lower intentions (Autio et al., 2001; Hmieleski and Corbett, 2006; Forlani and Mullins, 2000; Zhang et al., 2015). This may be explained by the fact that individuals who place an emphasis on risk aspects before taking action often lack proaction. Highly proactive people often make positive efforts to take actions to achieve their goals (Bateman and Crant, 1993; Zampetakis and Moustakis, 2006; Zampetakis et al., 2008; Neneh, 2019). Highly proactive people often have the ability to decide their own actions, willing to take risks to reap high benefits. This result also implies that, to promote entrepreneurship, students need help to be aware of business risk but the risk should not be overstated. Reducing cognitive risk also means promoting student initiative and entrepreneurship, two essential traits of entrepreneurs. Some studies of students also show that personal autonomy has a great influence on their entrepreneurial intentions (Zampetakis and Moustakis, 2006; Zampetakis et al., 2008; Neneh, 2019).

This study shows that we agreed with the majority of hypotheses in the TPB model. However, unlike previous studies, we have not found the direct impact of subjective norms on entrepreneurial intention. This finding shows that external influences such as relatives and friends have little influence on students' intentions or determination to entrepreneurship. This difference may arise from the shared character traits of students intending to start a new business, which are strong personality, independence, autonomy and their decisions tend to be less influenced by people around. The people close to them can influence their perception of a startup's likelihood of success and thereby influence their intentions. In other words, close people have an indirect influence on the intention to start a new business by influencing the perception of a startup's likelihood of success if they do it. Specifically, the estimated results from survey data showed the beta coefficient is negative (β < 0, p-value < 0.05). However, the model has a suppression phenomenon when the correlation coefficient between subjective norms and entrepreneurial intentions are positive (r > 0). Therefore, in this study, we believe that subjective norms only indirectly affect entrepreneurial intentions through perceived behavioral control. This again confirms that using the TPB model to predict entrepreneurship is appropriate.

The research results also indicated that the score of attitude towards entrepreneurship (ATT) and normative belief (BEL) is much higher than expectation (EXP), perceived risk (RIS) and perceived self-efficacy. This implies that students currently have a relatively high level of optimism about entrepreneurship and they are also heavily influenced by the people around them. At the same time, a high degree of optimism and the tendency to be influenced by other people is accompanied by low self-confidence and riskiness (low-risk perception). In other words, this result shows that students are optimistic about business, reckless but lack of confidence in themselves and their ability to conduct business activities. This may be because entrepreneurship training programs are not yet popular in Vietnam, especially for students.

### 5.2 Theoretical contributions

This study presents a more comprehensive framework than Krueger model (2000) to predict entrepreneurial behavior of which the first stage is entrepreneurial intentions. We have incorporated the perceived risks construct (RIS) and other individual characteristics into the model based on the theory of planned behavior (TPB). Although these constructs have already been mentioned in other previous research (Autio et al., 2001; Hisrich and Peters, 2012; Krueger et al., 2000a, Krueger et al., 2000b; Linan and Chen, 2009; Maes et al., 2014; Maresch et al., 2016), this is an attempt to combine them all in one model. We have also added control variables such as gender, major, place of residence before college and family employment. This research acts as a tool allowing universities to evaluate the impact of socio-psychological constructs on the perception of students and their entrepreneurial intentions.

### 5.3 Policy implications

The results of this research have important implications for universities to nurture and develop students' entrepreneurial intentions and entrepreneurial spirit to motivate them to start their businesses in the future when they have the opportunity. Through the research results, we suggest that universities should implement some measures as follows:

Firstly, engineering training programs should consider incorporating entrepreneurship and business courses in the programs. These courses may be offered in the form of orientation courses, extracurricular activities, an elective or compulsory subject to develop skills necessary for entrepreneurship. The coursework and study format should aim to develop the skills needed for entrepreneurial activities such as business planning skills, project management, teamwork skills or leadership skills. The combination of the business knowledge necessary for entrepreneurship and the ideas that come from technical disciplines may build up learners' confidence with entrepreneurship and boosting their entrepreneurial spirit.

Secondly, universities should develop more communication programs for female entrepreneurs. The image of powerful businesswomen and role models affecting the awareness and attitude of society is limited. This results in women having a lower intention of starting a business. Promoting communication on the image of businesswomen is also an activity to promote gender equality, encouraging female pupils and students to abolish the views of Confucian society on women's family roles and to motivate them to enhance their social roles.

Thirdly, universities should promote the implementation of programs that reduce the perceived risk of entrepreneurship among students. We suggest that universities may organize extracurricular activities such as entrepreneurship ideas competition or talk shows with successful entrepreneurs to promote the entrepreneurial spirit in students. Besides, universities may set up connection centers, business incubators to connect business projects with investors, universities with businesses to bring feasible business ideas into the market.

### 5.4 Limitations and directions for future research

As with any study, there are some limitations in our research that we suggest being addressed in the future. First, we use the entrepreneurial intentions construct instead of entrepreneurship behavior, which may make a difference from reality. Therefore, we suggest that future studies should consider combining entrepreneurship behavior construct to the research model to assess the effects of constructs on entrepreneurial intentions and from intention to behavior in reality of new opening business activities. Second, in this study, we assumed that business students have more entrepreneurial knowledge than engineering based on the fact that the business programs have many subjects related to entrepreneurship, business or management project. However, we did not analyze the duration and contents of these programs in much detail which may impact on entrepreneurial intentions. Although the aim of this study did not include how to analyze contents, duration or training method impacts entrepreneurial intentions. However, it would be an interesting topic for future study. To assess the impacts of different programs, a randomized trial experimental design would be preferred. Finally, our research is cross-sectional, therefore, causal inferences need to be further verified in the longitudinal studies in the future. More longitudinal studies in this field are encouraged to investigate developments over time, such as the relationship between entrepreneurial intentions and entrepreneurial behaviors (Kautonen et al., 2015). The role of perceived risk along other stages of the entrepreneurial process could also be discovered in greater depth.

## Declarations

### Author contribution statement

Kien Trung Dao and Trang Thi Thu Doan: Conceived and designed the experiments; Performed the experiments; Analyzed and interpreted the data; Contributed reagents, materials, analysis tools or data; Wrote the paper.

Tuan Anh Bui: Conceived and designed the experiments; Wrote the paper.

Tien Ngoc Dao AND Ha Thi Thu Le: Contributed reagents, materials, analysis tools or data; Wrote the paper.

Hoc Hieu Le: Conceived and designed the experiments; Contributed reagents, materials, analysis tools or data; Wrote the paper.

### Funding statement

This research did not receive any specific grant from funding agencies in the public, commercial, or not-for-profit sectors.

### Data availability statement

Data will be made available on request.

### Declaration of interests statement

The authors declare no conflict of interest.

### Additional information

No additional information is available for this paper.
